# A Year's Work at Guy's Hospital

**Published:** 1919-05-31

**Authors:** 


### THE HOSPITAL WORKS DEPARTMENT.

### A Year's Work at Guy's Hospital.

Whether a hospital has had a works department or not, during the war many matters of renovation or improvement have had to be deferred until a time of better supplies in labour and material. This was the case last year at Guy's Hospital, where in normal times extensive works are carried out by the hospital's own staff. The report of the architect and surveyor and the consulting engineer shows, however, that while there was necessarily a limitation in all structural repairs other than what was absolutely necessary for upkeep and maintenance, a great deal was carried out in the way of every-day repairs to such things as roofs, power-plant machinery, and urgent painting renovations.

### Coal Difficulties.

Had it not been for the hospital's foresight in maintaining a good supply of coal, there is no doubt that a serious position would have arisen which would possibly have entailed the shutting down of a large part of the work. It was not merely a question of rationing, to which a hospital, like other institutions and everybody else, had to adapt itself, as of the extreme difficulty in obtaining the allowed quantities. In respect of coal, 10 per cent. of the quantity requested was disallowed, 11½ per cent. of gas (and it must be remembered what wretched quality gas has recently been supplied), and 6½ per cent. of electricity. The average price of coal per ton in the last nine years has risen from 16s. 6d. in 1910 to 40s. 7d. in 1918, and the gross cost from £3,665 to £11,554. The most expensive year was 1917, when the average price was 41s. 4d.

### Electric Light and Power.

The hospital generates its own electricity, and last year used 350,544 units, which is near the average consumption of the last three years. The cost per unit (exclusive of interest and depreciation) has risen from 0.98 of a penny to 1.82. It would be interesting to see the figures when the two important items mentioned have been included, but the indication is that the enterprise of the hospital has been justified. We notice that a greatly increased number of carbon lamps were used in 1918, and fewer metallic filament, this doubtless because of the difficulty in getting the latter. The change, it must be noted, did not appreciably add to the number of units used, as the increase of 4,000 is neither here nor there in such a large undertaking.

### Upkeep and Supply of Equipment.

Pending the release from the Army of the surgical cutler, the department dealing with the repair of instruments has been closed, and 15,153 instruments were last year sent to outside firms. About 1,000 articles passed through the electro-plating shop, at a cost for labour and material of £61, and in other departments 800 crutches, 206 mattresses, 140 pillows, and a large number of miscellaneous articles were dealt with.

### The Staff of the Department.

The extent of the normal work of the works department at Guy's Hospital and the way in which it has been affected by the war is best illustrated by the table of cost shown in the annual report. In 1908 the expenditure on works of all kinds was £25,000. In the first year of the war the cost was £19,772, and last year it fell to £17,505. There were 127 men employed in August 1914, and there are only twenty-four on the staff at the present time. The hospital has made a half-pay allowance to twenty-three married men serving with the Forces.

It will be with some feeling of envy that the many hospital managers who have experienced continuous laundry difficulties during the war learn that the work of the laundry at Guy's has been carried on without interruption.

